# Pattern of injury in polytrauma compared to single limb related Lisfranc joint fractures

**DOI:** 10.1007/s00068-024-02702-9

**Published:** 2025-01-24

**Authors:** Chijioke Orji, Grace Airey, Darren Myatt, Lauren Greasley, Lucky Jeyaseelan, Isabella Drummond, Jitendra Mangwani, Khalis Boksh, Htin Kyaw, Hiro Tanaka, Mamdouh Elbannan, Lyndon Mason

**Affiliations:** 1 Liverpool Orthopaedic and Trauma Service, Liverpool University Hospitals NHS Foundation Trust, Liverpool, United Kingdom; 2 Institute of Health and Life Sciences, University of Liverpool, Liverpool, United Kingdom; 3 Barts Bone & Joint Health, The Royal London Hospital, Barts Health NHS Trust, London, United Kingdom; 4 Academic Team of Musculoskeletal Surgery, University Hospitals of Leicester NHS Trust, Leicester, United Kingdom; 5 Aneurin Bevan University Health Board, Newport, United Kingdom

**Keywords:** Lisfranc, Midfoot injury, Polytrauma, Single limb, Column injury

## Abstract

**Background:**

Midfoot fractures in polytrauma patients are often an underappreciated injury relative to their other major injuries sustained. In this study, our aim was to explore the mechanisms and patterns of injury in polytrauma related midfoot fractures as compared to single limb injuries.

**Setting:**

Multicentre observational study.

**Methods:**

Data was retrospectively collected from four centres (two major trauma centres and two trauma units) on surgically treated midfoot fracture dislocations between 2011 and 2021. Polytrauma was defined as a patient presenting with an Injury Severity Score (ISS) threshold of 15 or greater. Radiographs were analysed using departmental PACS. All statistics were performed using SPSS 26.

**Results:**

A total of 410 cases were included in the study. The rate of unstable midfoot injury was similar to simple falls, falls from height, crush injury, assault, sport and seizure. The only mechanisms that differ are a higher rate of midfoot injury in non-polytrauma patients undergoing a simple fall (19.71% vs. 6.78%) and higher rates of midfoot injury in polytrauma patients following motor vehicle collision (16.86% vs. 33.90%). Regarding patterns of injury, there was a significant increase in number of columns injured in polytrauma patients (polytrauma patient 3 column injury 77.97%, non-polytrauma patient 3 column injury 34.00%). There was no difference in the prevalence of central column injury (*p* = .623), although there were significantly more medial and lateral column injuries in the polytrauma group (*p* < .001 for both).

**Conclusion:**

Polytrauma related Lisfranc joint midfoot injuries have a higher prevalence of medial and lateral column injury than non-polytrauma Lisfranc joint midfoot injuries. Non-polytrauma injuries can, however, have an equally significant force involved as polytrauma patients, with over 50% occurring as the result of high velocity injury. A high index of suspicion should be maintained for midfoot injuries in high velocity mechanisms, regardless of other injuries sustained.

## Introduction

Midfoot injuries, encompassing a spectrum of damage from simple isolated fractures to complex fracture dislocations, present a significant challenge in orthopaedic trauma care due to their potential for long-term morbidity. Although midfoot fracture dislocations are relatively uncommon, they are potentially devastating injuries, with Lisfranc injuries having a reported incidence of 9.2/100 000 person-years while Chopart injuries have a reported incidence of 2.2/100 000 person-years [1]. Chopart-Lisfranc combinations are rare, with a reported incidence of 0.7/100 000 person-years [1].

Ponkilainen et al. reported in their study that low-energy trauma mechanisms caused just over half (54.9%) of the Lisfranc injuries, with high-energy trauma mechanisms causing just over a third (36.5%) of the injuries [1]. The aetiology of midfoot injuries are diverse, ranging from simple falls to high-energy impacts, with the latter often associated with polytraumatic events. Midfoot fractures in polytrauma patients are often an under-appreciated injury relative to their other major injuries sustained. Renniger et al. analysed the difference in injury characteristics between low-energy and high-energy midfoot injuries; however, this classification was based on the mechanism of injury and not severity [2].

Despite the acknowledged complexity of midfoot injuries and their management, there remains a gap in the literature regarding the comprehensive analysis of demographic characteristics and injury patterns between polytrauma and non-polytrauma patients. The primary aim of this study is to analyse the demographic and injury pattern differences between polytrauma and non-polytrauma related midfoot injuries.

## Methods

This was a historical cohort study of midfoot fracture/dislocations in four foot and ankle units in the UK, two level one major trauma centres and two level two trauma units. The collaboration between units was to enable collection of sufficient volume of cases. The protocol was reviewed by the research review board (Submission number 21 − 02) and was evaluated to be a service evaluation project and, therefore, did not require ethical approval. All surgically treated midfoot injuries were collected and analysed for potential inclusion into the study. Data was collected between January 2011 and August 2022. Inclusion criteria for this study were all tarsometatarsal joint injuries that had undergone surgical intervention. Exclusion criteria included patients under the age of 16 and mangled foot injuries.

**Fig. 1:**
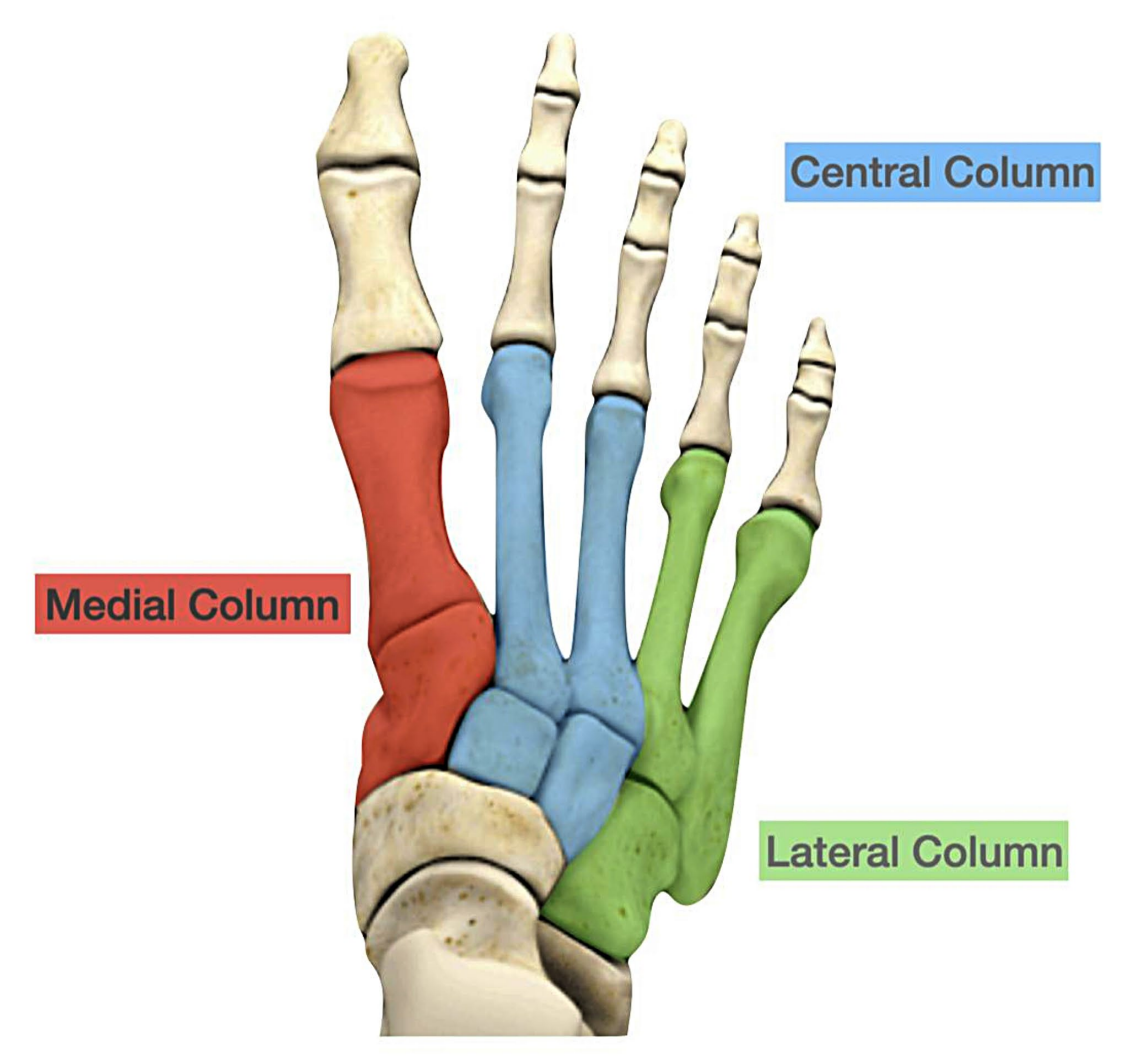
Schepers and Rammelt column classification

Patients’ medical records and radiological imaging were reviewed, and demographic data collected. The Computed Tomography (CT) images were reviewed by consultant radiologists who prepared diagnostic reports for each patient in each centre. These were not standardised. Authors from each centre then reviewed the reports and images to classify the injuries into columns. Radiological images were reviewed using departmental imaging software (Vue PACS, Carestream, Version 11.4.1.0324). Anonymised data was collected locally in each unit on prepared spreadsheets and sent to the lead centre for further analysis. The primary outcome of this study was to understand the prevalence of polytrauma in midfoot injuries. Polytrauma was defined as a patient presenting with an Injury Severity Score (ISS) threshold of 15 or greater [3]. Further data collected include demographics, presentation, and injury morphology. Column classification as described by Schepers and Rammelt, was determined using CT (Fig. 1) [4].

### Statistics

Continuous parametric data are presented as the mean and 95% confidence intervals, and dichotomous data as a cross-tabulation of frequencies and percentages. Statistical analysis was performed using student t test if continuous data was tested to be normal and Mann Whitney or Fisher’s Exact test if tested to be non-normally distributed. Binary data was tested using Chi-square. Significance was given to variables that reached *p* <.05. Statistical analysis was undertaken using SPSS statistics version 26 (IBM, New York, USA).

## Results

A total of 410 surgically treated unstable midfoot injuries were identified for further investigation. There were 59 cases recorded as polytrauma (14.39%). There were 237 (57.80%) males and 173 (42.20%) females in the entire cohort. The average age was 38.22 years (95% CI 36.67, 39.78). The polytrauma patients were younger, with an average age of 33.19 years (95% CI 29.46, 36.91) compared to 39.07 years (95% CI.

37.38, 40.77) in the non-polytrauma patient cohort. This was statistically significant (*p* = .007). There were significantly more males in the polytrauma cohort than in the non-polytrauma cohort (Table 1).

**Table 1:** Cross tabulation of gender and polytrauma. (N – number, % - percentage)

		Gender				Total	*P* value
		Male		Female			
		*N*	%	*N*	%		
Polytrauma	No	192	54.70	159	45.30	351	0.002
	Yes	45	76.27	14	23.72	59	
Total		237	57.80	173	42.20	410	

The mechanism of injury in both polytrauma and non-polytrauma cohorts is displayed in Table 2. The rates of injury by mechanism were similar across the two cohorts, including simple falls, falls from height, crush injury, assault, sport and seizure. The only mechanism that differed was a higher rate of midfoot injury in polytrauma patients following motor vehicle collisions (33.90% vs. 16.86%).

**Table 2:** Cross tabulation of mechanism and polytrauma. (N – number, % - percentage) % is shown as percentage of Polytrauma and Non-polytrauma cohorts by mechanism

		Non-Polytrauma		Polytrauma	
		*N*	%	*N*	%
Mechanism	Unknown	69	19.71	4	6.78
	Simple fall	112	32.00	16	27.12
	Fall from height	54	15.43	9	15.25
	Crush injury	31	8.86	6	10.17
	Assault	3	0.86	0	0.00
	Sport	21	6.00	4	6.78
	MVC	59	16.86	20	33.90
	Seizure	1	0.29	0	0.00
Total		350		59	

Analysis of the number of columns injured as per Schepers and Rammelt classification [4] showed an increasing number of columns injured, and thus complexities, in the polytrauma cohort compared to the non-polytrauma cohort (Table 3). Specifically looking at the columns injured, there was a significant increase in both medial and lateral column injury in the polytrauma patient cohort compared to the non-polytrauma cohort (Table 4). A total of 370 cases (90.24%) were recorded to have both bone and ligamentous injuries, with the rest purely ligamentous injuries. Only one patient in the polytrauma cohort (1.69%) had a purely ligamentous injury, compared to 10.86% in the non-polytrauma patient cohort (*p* = .027).

**Table 3:** Cross tabulation of number of columns and polytrauma. (N – number, % - percentage)

		Number of columns						Total	*p* Value
		1		2		3			
		*N*	%	*N*	%	*N*	%		
Polytrauma	No	88	25.14	143	40.86	119	34.00	350	< 0.001
	Yes	2	3.39	11	18.64	46	77.97	59	
Total		90	22.00	154	37.65	165	40.34	409	

**Table 4:** Cross tabulation of columns injury and polytrauma. (% - percentage) percentage given of positive column injury

		Medial column			Central column			Lateral column			Total
		No	Yes	%	No	Yes	%	No	Yes	%	
Polytrauma	No	137	214	60.97	17	334	95.16	167	183	52.14	351
	Yes	6	53	89.83	2	57	96.61	8	52	88.14	59
Total		143	267	65.12	19	391	95.37	174	235	57.32	410
Chi-Square		< 0.001			0.623			< 0.001			

Throughout the study, the majority of patients across all four treating units were treated with bridge plating (87.56%, *n* = 359). There was, however, a significant increase in the use of bridge plating (*p* = .03) and a significant decrease in the use of transarticular screws (*p* = .014) in the polytrauma cohort as compared to the non-polytrauma cohort. Fusion was rarely used across this study and was not used in any injuries categorised as ligamentous (Table 5).

**Table 5:** Category of surgical treatment by polytrauma category

		Polytrauma				Total	p value
		No		Yes			
		N.	%	N.	%		
Bridge plating	No	47	13.43%	2	3.45%	49	0.03
	Yes	303	86.57%	56	96.55%	359	
Transarticular screws	No	221	66.17%	47	82.46%	268	0.014
	Yes	113	33.83%	10	17.54%	123	
Fusion	No	337	97.12%	56	98.25%	393	0.628
	Yes	10	2.88%	1	1.75%	11	

## Discussion

**Fig. 2:**
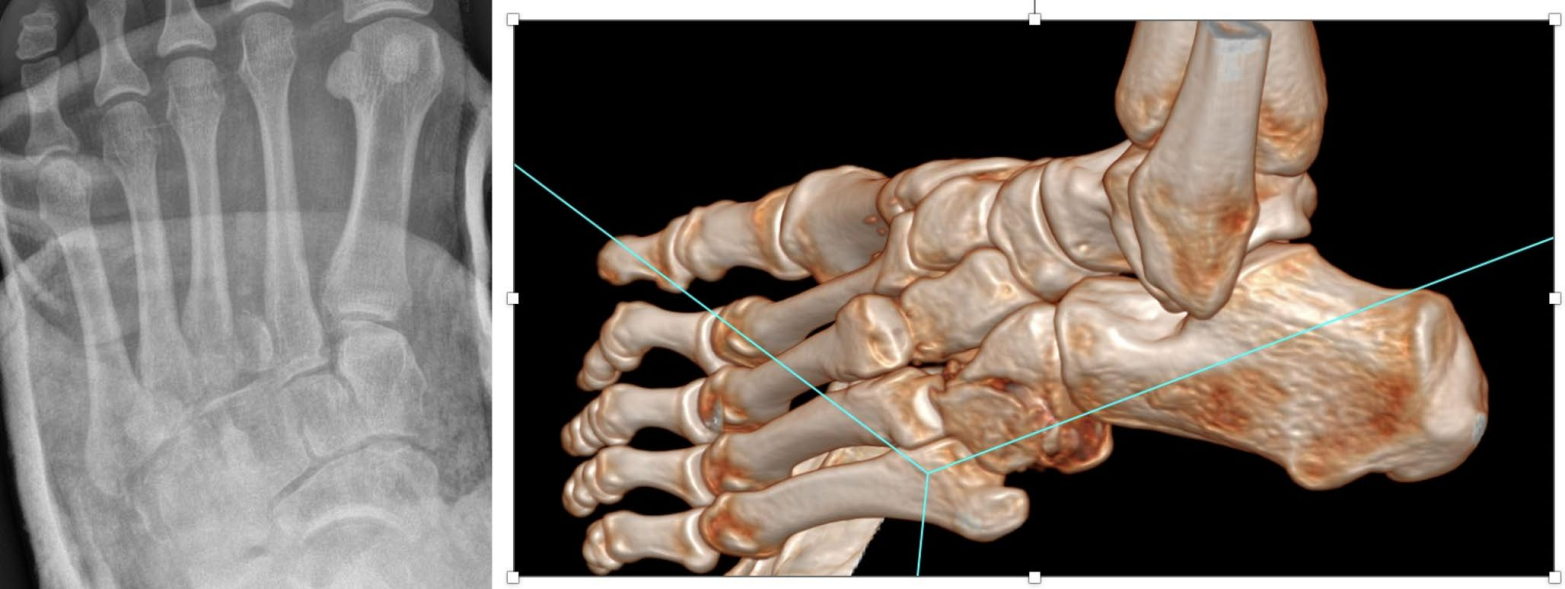
Radiograph and 3D surface rendering CT showing midfoot injury with affected central and lateral column in a polytrauma patient

**Fig. 3:**
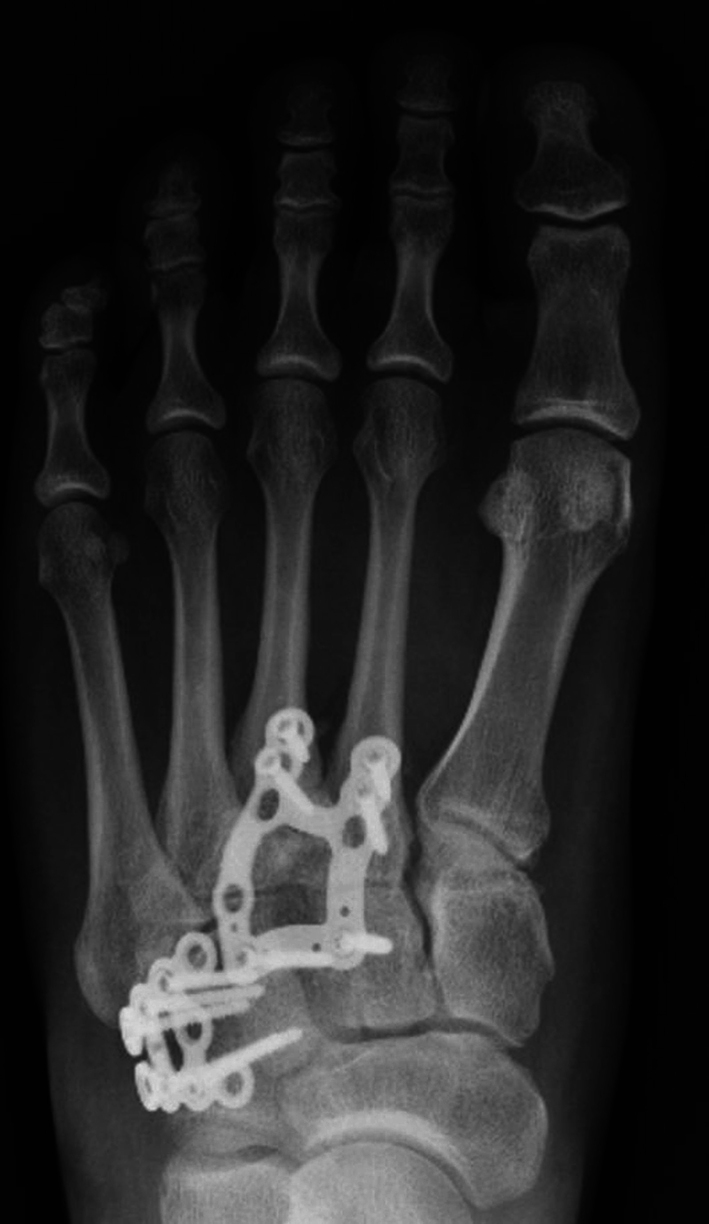
Anteroposterior radiograph of the patient in Fig. 1, treated with a central column bridge plate and cuboid buttress plate

The aim of this study was to analyse the demographic and injury pattern differences between polytrauma and non-polytrauma related midfoot injuries. It was identified that almost 15% of patients analysed in this study were the result of polytrauma. The polytrauma cohort was significantly younger and had a larger percentage of males. Regarding injury pattern, the findings revealed an increased number of injured columns in the polytrauma cohort compared to the non-polytrauma cohort, with a particularly significant increase in medial and lateral column injuries. Almost all the polytrauma patients had fracture dislocations (Figs. 2 and 3).

The rate of missed or delayed diagnosis for all cause midfoot injuries is relatively high, being reported in up to a quarter of patients undergoing basic radiographic imaging [5]. This can lead to poor long-term functional results if undermanaged [6]. Additionally, foot and ankle injuries in polytrauma patients have a missed rate of up to 39% due to distracting more severe and potentially life-threatening injuries requiring treatment [7]. Acknowledging the risk of misdiagnosis, a low threshold should be maintained to obtain advanced imaging of the foot (e.g. CT or MRI) in both polytrauma and non-polytrauma individuals who have clinical signs of midfoot injury. Tran and Thordarson reported in a study in polytrauma patients that an addition of a foot injury had a profound effect on the overall functional outcome of the patient [8]. In this study, we have found that the complexity of injury to the midfoot in polytrauma patients was significantly greater than in non-polytrauma patients, and care should be taken to identify the possible medial and lateral column injuries that arise more commonly in polytrauma individuals.

The observed increase in the number of injured columns in the polytrauma cohort could be attributed to several factors. First, the suspected higher energy nature of the trauma experienced by these patients might result in more extensive damage to the midfoot, increasing the likelihood of medial and lateral column injuries. This increase in the number of columns injured can result in a functional loss, with Lau et al. reporting the primary predictor of poor functional outcomes being the number of midfoot columns involved [9]. The significant increase in medial and lateral column injuries within the polytrauma cohort suggests that these patients may require more complex surgical interventions to address their injuries. Ponkilainen et al. reported no increase in the severity of injury between low energy and high energy mechanisms, contrary to the findings in this study; however, the inclusion of both trauma unit and major trauma patients in our study may mitigate selection bias [1]. These findings highlight the importance of prompt and accurate diagnosis of midfoot injuries in polytrauma patients and the need for specialised care to optimise outcomes.

The similarity in rates of injury by mechanism between polytrauma and non-polytrauma patients, other than motor vehicle collisions between the two cohorts, could suggest that the overall injury patterns may not be as distinct as would be expected. By definition, a polytrauma has an ISS greater than 15; thus, it is based on injury severity and the increased number of body locations where the injuries have been sustained [3]. Therefore, the mechanisms may not be exclusive but instead an indicator of the force sustained during injury. It is, therefore, important to have a low index suspicion for midfoot injury in polytrauma and, in turn, a low index of suspicion for injuries to other locations in midfoot injuries.

There are some limitations to consider when interpreting the results of this study. The sample size, although relatively large, may not be sufficient to generalise the findings to broader populations. The retrospective nature of the study might introduce selection bias or confounding factors that could impact the results. Additionally, our study only included surgically treated patients which may produce selection bias as we may have missed a much larger proportion of those treated conservatively with single limb injury especially considering polytrauma patients commonly have pan-scans. The Schepers and Rammelt classification may not capture all relevant aspects of midfoot injury patterns and severity [4]. Similarly, treating ISS as a dichotomous variable may affect comparisons between groups, especially the patients with ISS near 15.

## Conclusions

Polytrauma related midfoot injuries have a higher prevalence of medial and lateral column injury than non-polytrauma midfoot injuries. Non-polytrauma midfoot injuries can, however, have an equally significant force involved as polytrauma patients, with over 50% occurring as the result of high velocity injury. A high index of suspicion should be maintained for midfoot injuries in high velocity mechanisms, regardless of other injuries sustained.

## Data Availability

No datasets were generated or analysed during the current study.
